# A Protective Role of the NRF2-Keap1 Pathway in Maintaining Intestinal Barrier Function

**DOI:** 10.1155/2019/1759149

**Published:** 2019-06-26

**Authors:** Zhiyong Wen, Weihua Liu, Xing Li, Weiguo Chen, Zhice Liu, Jianbo Wen, Zhiping Liu

**Affiliations:** ^1^Gannan Medical University, Ganzhou, Jiangxi 341000, China; ^2^Xingguo People's Hospital, Xingguo, Jiangxi 342400, China; ^3^Department of Gastroenterology, Pingxiang People's Hospital, Pingxiang, Jiangxi 337000, China; ^4^School of Basic Medicine, Gannan Medical University, Ganzhou, Jiangxi 341000, China

## Abstract

Nrf2 (NF-E2-related factor 2) is a master regulator of cellular oxidative levels against environmental stresses. Nrf2 induces the expression of metabolic detoxification and antioxidant enzymes to eliminate reactive oxygen species (ROS). The gastrointestinal tract is a key source of ROS. Intestinal barrier is critical to maintain the healthy steady state of the human gastrointestinal tract. Nrf2 has been shown to play important roles in maintaining the integrity of intestinal mucosal barrier. Here, we made a systematic review on the roles of Nrf2 in maintaining intestinal barrier, including the following: (1) NRF2 reduced intestinal mucosal injury by suppressing oxidative stress; (2) NRF2 decreased intestinal inflammation by inhibiting the inflammatory pathway; (3) NRF2 affected intestinal tight junction proteins and apoptosis of cells to regulate intestinal permeability; (4) NRF2 affected T cell differentiation and functions; (5) the crossregulation between the KEAP1-NRF2 pathway and autophagy controlled intestinal oxidative stress.

## 1. Introduction

The intestinal environment of the human body is very complex. Intestinal barrier plays a very important role in resisting the external environmental stress. The intestinal mucosa consists of several elements to form the physical and immunological defense barrier. These elements mainly include the outer mucus layer with the intestinal commensal bacteria, antimicrobial peptides (AMPs), and secretory immunoglobulin A (sIgA) molecules, the central single cell layer with specialized epithelial cells, and the inner lamina propria which contains innate and adaptive immune cells such as T cells, B cells, macrophages, and dendritic cells [1]. The intestinal epithelium is a single layer of cells lining the gut lumen, which not only prevents the passage of harmful intraluminal entities including foreign antigens, microorganisms, and their toxins, but also works as a selective filter to allow the translocation of essential dietary nutrients, electrolytes, and water from the intestinal lumen into the circulation. The intestinal epithelium mediates selective permeability by transepithelial/transcellular and paracellular pathways [2].

Intestinal permeability is regulated by multiple factors including exogenous factors, epithelial apoptosis, cytokines, and immune cells [2]. In addition, intestinal lumen contains millions of microorganisms. Intestinal epithelial cells (IECs) must tolerate the presence of the commensal microbiota bacteria and thus must not respond to their products, but they still protect the intestinal mucosa from potentially harmful dietary antigens and invading pathogens. IECs express a variety of innate immune receptors, such as Toll-like receptors (TLRs), to detect microbes and endogenous danger signals. Intestinal intraepithelial lymphocytes (IELs) reside between IECs and participate in the formation of the intestinal mucosal barrier. Upon pathogen invasion, deregulation of mucosal immunity or damage to IELs causes the disruption of intestinal homeostasis to exacerbate inflammation [3].

Nrf2 (NF-E2-related factor 2) is a master regulator of cellular responses against environmental stresses. Nrf2 induces the expression of detoxification and antioxidant enzymes and Keap1 (Kelch-like ECH-associated protein 1), an adaptor subunit of cullin 3-based E3 ubiquitin ligase. Under the stress condition, Keap1 induces Nrf2 translocation from the cytoplasm to the nucleus and thus activates the expression of multiple target genes [4]. The target genes of Nrf2 include the genes encoding antioxidant enzymes, drug-metabolizing enzymes and transporters, and heme and iron metabolic enzymes [5]. The expression level of Nrf2 is particularly high in the detoxification organs or tissues which directly counter the environment, such as the intestine, lung, and choroid plexus of the brain in a mouse embryo [4].

Recent studies have found that the Nrf2-Keap1 pathway participates in many other cellular protective mechanisms besides countering oxidative stress, including the regulation of inflammatory pathways and tight junction (TJ) proteins in intestinal barrier. In this review, we will summarize the roles of the Nrf2-keap1 pathway in maintaining intestinal barrier through regulating oxidative stress, intestinal inflammation, intestinal permeability, and T cell activation and autophagy.

## 2. NRF2 Reduced Intestinal Mucosal Injury by Controlling Oxidative Stress

Overproduction of reactive oxygen species (ROS), the byproducts of normal cellular metabolism, will damage intestinal mucous barrier. In this section, we will introduce that NRF2 activation will significantly suppress ROS generation, enhance cell survival, and improve cell redox state in intestinal epithelial cells. This effect can be observed in many experimental models of intestinal barrier injury.

ROS include radical compounds such as superoxides (O2^−^), hydroxyl radicals (-OH), lipid hydroperoxides, and reactive nonradical compounds including singlet oxygen, hydrogen peroxide (H_2_O_2_), hypochlorous acid (HOCl), chloramines (RNHCl), and ozone (O_3_) [6]. Moderate ROS have beneficial effects on several physiological processes including killing of invading pathogens, wound healing, and tissue repair processes. However, overproduction of ROS results in oxidative stress and causes disruption of homeostasis and oxidative tissue damage to the human body. It causes the damage of cellular lipids, proteins, and DNA, increases cellular swelling, and decreased cell membrane fluidity. The gastrointestinal (GI) tract is a key source of ROS, and ROS are involved in many GI diseases. Excessive oxidative stress will result in intestinal inflammation and apoptosis of intestinal mucous epithelium, which further damages intestinal mucosa barrier [7].

The Nrf2-Keap1 pathway plays a key role in resisting intestinal mucosal injury. *Nrf2-deficient* mice treated with DSS for 6 days had substantially higher levels of lipid peroxidation, including lipid peroxidation products (malondialdehyde and 4-hydroxyalkenals), in colons than WT mice. Consistently, in Caco-2 cells, the nuclear translocation of Nrf2 significantly suppressed ROS generation and enhanced cell survival and glutathione S-transferase P1 (GSTP1) expression [8]. ROS is one of the pathogenic factors responsible for intestinal injury in ulcerative colitis (UC). The expression level of *Nrf2* gene increased in UC patients by quantitative PCR in inflamed colonic tissues compared to the control group. At the same time, Nrf2 expression could elevate the mRNA expressions of glutathione S-transferase alpha 4 (GST-A4, against oxidative damage) in the colonic samples of the UC patients [9]. Activation of Nrf2 by biogenci nanoselenium (BNS) results in potent protection against diquat which induces an epithelial barrier injury effect. It reduced oxidative stress by reducing cell apoptosis and improved the cell redox state in dose-dependent and time-dependent manners. However, knockdown of *Nrf2* reversed the protection effect of BNS [10].

The protective effect of Nrf2 in maintaining the barrier has been proved in various experimental models, including *Salmonella typhimurium* infection [11], dextran sodium sulfate-induced colitis [11–13], intestinal ischemia-reperfusion [14, 15], aspirin/NSAID-induced vascular damage [16], intestinal burn [17], severe sepsis [18], and traumatic brain injury-induced intestinal mucosa damage and epithelial barrier dysfunction [19] (Table 1).

## 3. NRF2 Suppresses Intestinal Inflammation by Inhibiting Inflammatory Pathway

NRF2 activation can enhance the host protection from ROS stress. More recent studies have also shown that intestinal Nrf2 activation can suppress the NF-*κ*B pathway. But the activation of the NRF2-Keap1 pathway may reduce inflammation by decreasing ROS generation. In this situation, we also introduce that Keap1 dissociated from Nrf2 suppresses the ubiquitination and degradation of I*κ*B, thus inhibiting the activation of NF-*κ*B. Keap1 may indirectly regulate TANK-binding kinase 1 (TBK1) through IKK*β*.

**Figure 1:**
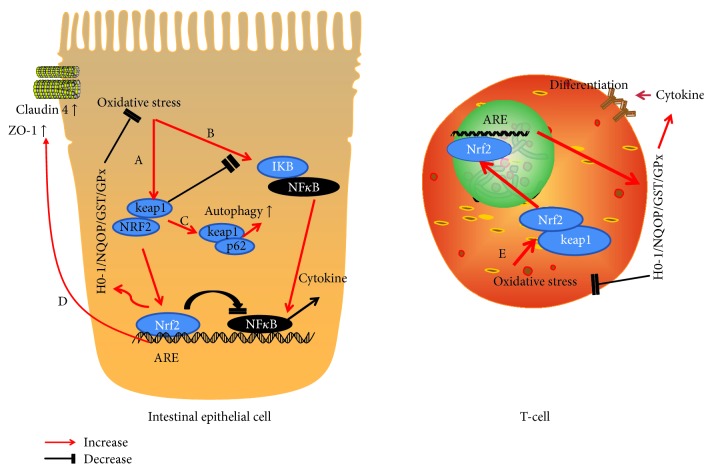
The pathway of Nrf2 in the protection of intestinal mucosal barrier. (A) Keap1 induces the translocation of Nrf2 from the cytoplasm to the nucleus and activates the expression of a wide array of target genes. As a result, the intestinal oxidative injury decreases. (B) The activation of Nrf2 can inhibits inflammatory pathway and reduces cytokine production. Dissociated Keap1 suppresses the ubiquitination and degradation of I*κ*B, thus inhibiting the activation of NF-*κ*B. (C) The binding of Keap1 to p62 causes the autophagy of ubiquitinated proteins. Keap1 participates in the regulation of the Nrf2-keap1-P62-autophagy pathway as a bridge. These two antioxidant pathways coordinate to maintain intestinal barrier function under different physiological and pathological conditions. (D) The activation of Nrf2 increases the expression of the tight junction proteins ZO-1 and occludin. (E) The activation of Nrf2 induces T cell activation and cytokine production.

ROS production can induce proinflammatory responses through activating redox-sensitive transcription factors such as NF-*κ*B and AP-1 and upregulating kinases including MAPKs (p38, ERK, and JNK) and PI3K. Overt inflammation, such as inflammatory bowel diseases (IBD), can make severe damage to intestinal barrier function [20]. ROS generation can lead to the activation of immune cells and the development of chronic inflammation. However, chronic inflammation can also aggravate ROS generation, which results in a vicious circle [7]. It is suggested that decreased ROS generation can reduce inflammation. A number of studies have demonstrated that there is an interplay between the Nrf2 and NF-*κ*B pathways. In order to test it, *Nrf2-deficient* mice were treated with dextran sulfate sodium (DSS) to induce colitis. *Nrf2-deficient* mice had an increased inflammation level compared to wild-type (WT) mice. Moreover, the expression of proinflammatory mediators, including IL-1*β*, IL-6, TNF-a, iNOS, and COX-2, increased significantly in *Nrf2^−/−^* mice [21]. In another study, THP-1 cells with Nrf2 gene silencing were treated with lipid-associated membrane proteins (LAMPs) to investigate the effect of Nrf2 on inflammatory cells (Figure 1). The results showed that IL-6, IL-8 iNOS, and Cox-2 gene expression levels and the release of NO and PGE2 increased after Nrf2-silenced [22]. One study investigated the roles of Nrf2 in traumatic brain injury-induced gut barrier dysfunction. Nrf2 not only influenced NF-*κ*B activity and the production of inflammatory cytokines (TNF-*α*, IL-1*β*, and IL-6) but also affected ICAM-1 gene expression, intestinal permeability, and plasma endotoxin level [23]. However, NF-*κ*B activation could be attenuated by diverse Nrf2 activators, such as dh404. In order to observe uremia-associated intestinal inflammation, CKD was induced via 5/6 nephrectomy in Sprague–Dawley (SD) rats and dh404, a Nrf2 activator, was used to study the effects of systemic Nrf2 activation. Consistently, Nrf2 activation by dh404 attenuated colonic inflammation, decreased P-I*κ*B*α*level, and decreased gene expression of COX-2, MCP-1, iNOS, and gp91 compared to SD rats without dh404 treatment [24].

In conclusion, Nrf2 activity can attenuate intestinal inflammation; however, it remains unclear whether the reduction in inflammation is due to the suppression of ROS production by Nrf2 activation or to suppression of inflammatory pathways directly by Nrf2. Some studies have showed that KEAP1, an oxidative stress sensor, seems to be involved in regulating the inflammation. Under normal conditions, the Keap1/Cul3-Rbx1 complex facilitates the degradation of Nrf2 constantly. However, when a cell encounters oxidative or electrophilic stress, Nrf2 dissociates from the complex and then translocates into the nucleus, where it activates antioxidant and cytoprotective gene expression. In order to observe the role of Nrf2 in the development of inflammation, primary human macrophages with the *KEAP1* gene knowckdown were infected with *Mycobacterium avium* to induce inflammation. The ROS generation and Nrf2 activation were both elevated. But it was found that knockdown of *Keap1* increased the gene expression of inflammatory cytokines, such as TNF-*α*, IL-6, IL-1*β*, CXCL10, and IFN-*β* mRNA levels. What is more, Keap1-Cul3-Rbx1 was shown to be specifically responsible for ubiquitin-mediated degradation of IKK*β* which will result in the termination of IKK activity, and the Keap1 also indirectly regulated TBK1 through IKK*β* [25]. In another study, with the activation of Nrf2, Keap1 is associated with SQSTM1/p62-bodies; adaptor proteins are related to autophagy and contribute to the decrease of CXCL10 expression [26]; therefore, more studies are required to clarify whether the Nrf2 effect is related to the inflammatory pathway or ROS production, or both.

## 4. NRF2 Regulates Intestinal Permeability by Affecting Intestinal Tight Junction Proteins and Cell Apoptosis

Tight junction proteins are important for the permeability of the intestinal epithelial barrier. Nrf2 has been shown to affect epithelial tight junction protein expression in animal models. In this section, we can conclude that the Nrf2 activation in intestinal barrier can influence the expression of tight proteins and apoptosis of IECs.

Permeability of the intestinal epithelial barrier is controlled by several multiprotein adhesive complexes (junctions) located along the lateral surface of contacting epithelial cells. These structures include the most apical tight junctions (TJs), subjacent adherent junctions (AJs), and desmosomes [27]. There are three main families of TJ proteins: the claudin family, the occludin family, and the IgG-like family of junctional adhesion molecules (JAMs) [28]. The incorporation and association of the three TJ proteins require local clustering of scaffolding proteins, an important group of ZO proteins, such as ZO-1, ZO-2, and ZO-3 [27]. Claudins are the main determinants of barrier properties of the TJs [28].

A study found that, in a traumatic brain injury-induced intestinal mucosa damage model, intestinal permeability and plasma endotoxin level of *Nrf2^−/−^* mice increased in comparison with those of wild-type mice [23]. Another study showed that the ERK/Nrf2/HO-1 signaling pathway mediated mitophagy-decreased intestinal oxidative stress, while the expression of the tight junction proteins zona occludens 1 (ZO-1) and occludin was enhanced. Furthermore, autophagy activators or inhibitors altered the expression of these tight junction proteins [19]. In a colonic inflammation model induced by chronic kidney disease (CKD), the Nrf2 activator, dh404, restored the levels of epithelial tight junction proteins including ZO-1, occludin, and claudin-1 [24].

One study found that Nrf2 was activated in esophageal epithelial cells in human gastroesophageal reflux. In order to directly study the role of Nrf2 in intestinal barrier, the *Nrf2* gene was knocked down in mice to observe the impairment of esophageal epithelium barrier. It was found that Cldn4, but not Cldn1, was downregulated in the cytoplasm and cell membrane of esophageal epithelial cells of *Nrf2^−/−^* mice compared to those of wild-type mice. Further analysis revealed that an activated Nrf2 bone conserved to the binding site of Ocld1 and altered the expression of Cldn4. However, Cldn1 lacked such similar sites [29].

In addition, other studies showed the activation of the Nrf2-Keap1 pathway not only influenced the tight junction proteins but also protected the intestine epithelial cells from apoptosis [15] and controlled the overproliferation of intestinal stem cells to maintain intestinal homeostasis [30].

## 5. NRF2 Regulates Intestinal Immune Function by Affecting T Cell Differentiation

Th1/Th2 differentiation is a critical process for host immunological defense in the intestine. Activation of Th1 cells eliminates intracellular pathogens by activating macrophages and releasing various cytokines. Th2 cells stimulate B cells to secrete antibodies and play important roles in host defense against worm infection. Activation of Nrf2 may alter Th1/Th2 balance demonstrated by changes in the cytokine production. This effect may be achieved through modulation of AP-1 and NF-*κ*B activity [31].

The abundance of innate and adaptive immune cells resides closely with trillions of commensal microorganisms in the human gastrointestinal tract, which requires barrier and regulatory mechanisms that restrict host-microbial interactions and maintains tissue homeostasis. Th1/Th2 differentiation is a critical process in tailoring adaptive immune responses against commensal bacteria [32].

A study showed that the activation of Nrf2 may alter the Th1/Th2 balance. Nrf2 activation by tBHQ markedly decreased IFN-*γ* production markedly in splenocytes stimulated with CD3/CD28 in mice. Furthermore, Nrf2 activation promoted Th2 differentiation of CD4^+^T cells, inhibiting Th1 differentiation shown by corresponding changes in Th1/Th2 cytokine productions [33]. Another study found that Nrf2 activated by tBHQ inhibited IL-2 production and decreased the expression of CD25, an early T cell activation marker, in human Jurkat T cells. In addition, it was found that activated Nrf2 induced the nuclear translocation of the transcription factors AP-1 and NF-*κ*B but not the c-fos and c-jun pathway and decreased calcium influx. It suggested that Nrf2 activation suppressed several key events of early T cell activation [31]. Another study focused on the role of Nrf2 activation in acute graft-versus-host disease (aGVHD). It was found that dimethyl fumarate (DMF), a Nrf2 activator, could inhibit alloreactive T responses by suppressing cell proliferation and inducing cell apoptosis in *vitro*. Furthermore, Nrf2 activation promoted donor Treg development and reduced effector function of alloreactive T cells, thus leading to the alleviation of aGVHD severity [34].

A more recent study found that NRF2 activation by different inducers has different effects on the early T cell differentiation. Nrf2 activation by tBHQ or CDDO-IM (the imidazolide derivative of the triterpenoid) has little effect on the induction of CD25 and CD69 in activated mouse splenocytes. The activation of some T cell secretion endpin cytokines are independent on the Nrf2 pathway, so it may be inaccurate to assess whether Nrf2 influenced T cell differentiation by only detecting cytokine changes or not [35].

Other studies also showed that *Nrf2*^−/−^ mice had increased plasma endotoxin levels compared to wild-type mice in intestinal barrier injury [23]. Although Nrf2 has effects on T cell activation, the exact mechanism remains largely unknown. More studies are required to fully understand the effect of NRF2 on the activity of intestinal immune cells in intestinal barrier.

## 6. The Effect of Crossregulation between the KEAP1-NRF2 Pathway and Autophagy on Intestinal Oxidative Stress

Autophagy is the catabolic process of delivering cytoplasmic constituents, organelles, and infectious agents to the lysosome for degradation. Autophagy is crucial in maintaining antimicrobial defense, epithelial barrier integrity, and mucosal intestinal immune response [36]. Together with the Nrf2-dependent regulation of antioxidant responses, the autophagy-lysosomal pathway provides additional defense mechanisms against cellular oxidative stress by removing selectively misfolded or damaged intracellular proteins and organelles.

But mechanisms how the autophagy pathway responds to oxidative stress signals are still largely unclear. Some studies have suggested that mTOR and PI3K mediate the autophagy responses to oxidative stress and thereby attenuate or reverse the injury caused by oxidative stress [37, 38].

Recent studies indicate a strong association between the Keap1-Nrf2 antioxidative pathway and autophagy. The p62/SQSTM1 protein (hereafter referred to as p62) is located in the sites of autophagosome formation and can be associated with both the autophagosome localizing protein LC3 and ubiquitinated proteins. Sequestosome1 (SQSTM1)/p62 is a protein of multifunctional adapter that accumulates following autophagy inhibition and can serve as a diagnostic marker for human autophagic vacuolar myopathies (AVMs). One study found that autophagy inhibition in AVWs led to the sequestration of the Nrf2 pathway inhibitor Keap1 into P62-positive protein, which aggregates the Nrf2-mediated stress response pathway [39]. It was also found that knockdown of *Nrf2* reduced the cellular level of p62. In addition, knockdown of *p62* reduced the cellular level of *Nrf2* [40]. By developing a splicing variant of p62/Sqstm1 pre-mRNA, one study suggested that the variant without the Keap1-interacting region (KIR) increased the amount of Keap1 and enhanced ubiquitination of Nrf2, thereby suppressing the induction of Nrf2 target genes [41]. Genetic ablation of Keap1 led to the accumulation of ubiquitin aggregates and defective activation of autophagy, suggesting that Keap1 binding to p62 may also be involved in p62-mediated autophagy of ubiquitinated proteins [42]. So Keap1 worked as a bridge participating in the regulation of the p62-keap1-NRF2 pathway. P62 phosphorylated in an mTORC1-dependent manner implied the coupling of the Keap1-Nrf2 system to autophagy. Phosphorylation of p62 increased its binding affinity to Keap1 and the subsequent induction of the expression of Nrf2 target genes [43].

Therefore, dysregulation of the P62-Keap1-Nrf2 pathway may damage intestinal barriers. In an *Atg5* conditional knockout (cKO) mouse model, intestinal epithelial injury induced by indomethacin significantly decreased in Atg5-cKO mice compared to WT mice. The attenuation of intestinal injury includes intestinal lesions and ulcer, epithelial apoptosis, mitochondrial membrane, and ROS toleration. Furthermore, the activity of the ERK/Nrf2/HO-1 pathway significantly increased which contributed to the decrease of intestinal injury after indomethacin (IM) treatment in Atg5-cKO mice [44].

These two antioxidant pathways seem to coordinate their responsibilities in maintaining intestinal barrier function under different physiological and pathological conditions, but more studies are needed to investigate this crossregulation.

## 7. Conclusion

In recent years the incidence of intestinal disease, such as IBD, has increased significantly due to changes in dietary habits and other environmental factors [45]. It is crucial to understand the mechanism of intestinal barriers for the development and treatment of intestinal disease. In this review, we elucidated the following important roles of Nrf2 in maintaining intestinal barrier. First, activation of the Nrf2 pathway can reduce intestinal barrier injury in different mouse disease models by suppressing ROS generation and enhancing cell survival and transcription of antioxidant target genes. Second, suppression of ROS generation by Nrf2 can decrease intestinal inflammation. In addition, Keap1 seems to be involved in the regulation of the inflammation pathway. Knockdown *Keap1* increased the production of NF-*κ*B inflammatory cytokine [25]. Furthermore, Keap1 serves as a bridge between autophagy and Nrf2 in the p62-keap1-Nrf2 pathway to maintain cellar homeostasis [42]. Third, Nrf2 activation not only influenced the expression of tight junction proteins but also protected the intestine epithelial cells from apoptosis and controlled the overproliferation of intestinal stem cells to enhance intestinal homeostasis. Finally, Nrf2 may have an effect on the activation of T cells.

There are substantial interests in developing Nrf2 inducers for therapeutic use. For example, the methyl ester derivative (CDDO-Me) has been found in many clinical trials to treat a variety of diseases, including chronic kidney disease, type 2 diabetes, liver dysfunction, and certain cancers. Another attractive Nrf2 inducer is dimethyl fumarate, which has been approved by the FDA for the treatment of relapsing multiple sclerosis [46]. However, the lethality of Keap1 knockout mice indicates that constitutive activation of Nrf2 in the upper digestive tract tissues can result in serious adverse effects. Furthermore, some studies have demonstrated a link between oncogenesis and mutations in the Keap1-Nrf2 pathway that result in the constitutive activation of Nrf2 [5]. Anyway, Nrf2 becomes a potential target in the treatment of multiple intestinal diseases induced by mucosal barrier injury.

## Figures and Tables

**Table 1:** The roles of Nrf2 in the protection of intestinal barrier in various disease models.

Model	The effect of activating Nrf2	Reference
S. typhimurium infection	Oxidative stress and colitis decreased.	Theiss et al. [11]
Dextran sodium sulfate-induced colitis model	Numbers of aberrant crypt foci decreased, and colonic proinflammatory cytokine, myeloperoxidase activity, 3-nitrotyrosine immunoreactivity, and lipid peroxidation decreased. Aconitase activity increased.	Osburn et al. [12]
Burn-injured model	Damage of gut structure, intestinal permeability, and inflammatory cytokines (IL-6, IL-1*β*, MCP-1, intercellular adhesion molecule, and vascular cell adhesion molecule) decreased. NAD(P)H dehydrogenasequinine-1 and glutamate-cysteine ligase modifier subunit increased.	Chen et al. [17]
Intestinal ischemia reperfusion injury model	Mucosal 15-F2t-isoprostane was reduced. Endogenous antioxidant superoxide dismutase activity was elevated.	Han et al. [14]
Rat liver transplantation model	HO-1 expression was elevated. Tight junction function was restored.	Chi et al. [15]
Traumatic brain injury model	Apoptosis-mediated intestinal epithelial cell damage was diminished. Intestinal permeability was improved.	Liu et al. [19]
Aspirin/NSAID-induced vascular damage model	NSAID-induced vascular permeability was reduced. Mucosal MPO activity increased. Anaerobic enterobacterial count decreased.	Yanaka et al. [16]
Severe sepsis model	Injuries caused by oxidative stress and inflammation were alleviated. HMGB1 levels was reduced, but HO-1 levels increased.	Yu et al. [18]
